# Abnormal salience signaling in schizophrenia: The role of integrative beta oscillations

**DOI:** 10.1002/hbm.23107

**Published:** 2016-02-08

**Authors:** Elizabeth B. Liddle, Darren Price, Lena Palaniyappan, Matthew J. Brookes, Siân E. Robson, Emma L. Hall, Peter G. Morris, Peter F. Liddle

**Affiliations:** ^1^ Institute of Mental Health, School of Medicine, University of Nottingham Nottingham United Kingdom; ^2^ Sir Peter Mansfield Imaging Centre, School of Physics and Astronomy University of Nottingham, University Park Nottingham United Kingdom

**Keywords:** schizophrenia, magnetoencephalography, neuroimaging, electrophysiology, attention

## Abstract

Aberrant salience attribution and cerebral dysconnectivity both have strong evidential support as core dysfunctions in schizophrenia. Aberrant salience arising from an excess of dopamine activity has been implicated in delusions and hallucinations, exaggerating the significance of everyday occurrences and thus leading to perceptual distortions and delusional causal inferences. Meanwhile, abnormalities in key nodes of a salience brain network have been implicated in other characteristic symptoms, including the disorganization and impoverishment of mental activity. A substantial body of literature reports disruption to brain network connectivity in schizophrenia. Electrical oscillations likely play a key role in the coordination of brain activity at spatially remote sites, and evidence implicates beta band oscillations in long‐range integrative processes. We used magnetoencephalography and a task designed to disambiguate responses to relevant from irrelevant stimuli to investigate beta oscillations in nodes of a network implicated in salience detection and previously shown to be structurally and functionally abnormal in schizophrenia. Healthy participants, as expected, produced an enhanced beta synchronization to behaviorally relevant, as compared to irrelevant, stimuli, while patients with schizophrenia showed the reverse pattern: a greater beta synchronization in response to irrelevant than to relevant stimuli. These findings not only support both the aberrant salience and disconnectivity hypotheses, but indicate a common mechanism that allows us to integrate them into a single framework for understanding schizophrenia in terms of disrupted recruitment of contextually appropriate brain networks. *Hum Brain Mapp 37:1361‐1374, 2016*. © 2016 Wiley Periodicals, Inc.

## INTRODUCTION

We call an event “salient” when it catches our attention. Its salience can be due to its sensory properties (“bottom up” salience), or its relevance to current concerns (“top–down” salience) [Corbetta and Shulman, 2002]. Both tend to produce a phasic increase in dopamine in circuits implicated in associative learning [Schultz et al., 1997], binding the event to its current context, establishing causal links, and facilitating prediction. Kapur [2003] hypothesized that psychosis arises from “aberrant salience” in which excess dopamine activity leads to the attribution of unwarranted salience to stimuli that would not ordinarily be regarded as significant, leading to a pathologically heightened sense of the significance of everyday occurrences, perceptual distortions, and delusional causal inferences.

Palaniyappan and Liddle [2012] extended Kapur's concept of abnormal salience to embrace not just the hallucinations and delusions of psychosis but the disabling disruptions to cognition and volition of chronic schizophrenia. They put forward the concept of “proximal salience,” with a hypothesis involving the role of the anterior insula in salience processing. The anterior insula is a key node of a “salience network” [Menon and Uddin, 2010; Seeley et al., 2007; Sridharan et al., 2008], and implicated in integrating salient sensory and interoceptive information with representations of goals and outcomes [Augustine, 1996; Bossaerts, 2010; Kurth et al., 2010]. Structural and functional abnormalities of the anterior insula are implicated in mental health disorders [Nagai et al., 2007], and a meta‐analysis of structural brain imaging studies in schizophrenia by Glahn et al. [2008] identified the insula as the site of the most consistent grey matter abnormalities. Citing this evidence, Palaniyappan and Liddle [2012] postulated that in schizophrenia, disruption to insula connectivity results in inappropriately assigned “proximal salience,” defined as a property attained by “an event, such as an externally generated sensation, a bodily sensation or a stimulus‐independent thought… when it generates a momentary state of neural activity within the salience network that results in updating of expectations and, if warranted by the context, initiates or modifies action.” Inappropriately assigned proximal salience, they postulated, leads not only to the perceptual and cognitive distortions of acute psychosis but also, via disrupted information processing, to the symptoms of disorganization, and, via disrupted goal‐setting, to the negative symptoms of psychomotor poverty.

This broader disrupted salience connectivity model is supported by three recent fMRI studies [Manoliu et al., 2014; Moran et al., 2013; Palaniyappan et al., 2013] that indicate that the directional influence of the insula on the dorsolateral prefrontal cortex is impaired in schizophrenia. The model thus not only extends the concept of aberrant salience in schizophrenia to account for disruption to cognition and volition but also links aberrant salience to the long‐standing concept of schizophrenia as a dysconnection syndrome [Fitzsimmons et al., 2013; Fornito et al., 2012; Friston and Frith, 1995].

In order to investigate brain networks involved in the assignment of proximal salience in health and in schizophrenia, we developed a Relevance Modulation (RM) task in which behavioral salience was manipulated by alternating relevant stimuli with irrelevant stimuli that were visually identical, and in which only a minority of relevant stimuli required an overt behavioral response [Brookes et al., 2015, 2012]. This task enables us to isolate solely top–down effects of task relevance from bottom–up sensory salience common to both conditions, as well as from signal associated with the motor response itself.

Although functional cortical networks have hitherto largely been delineated in fMRI studies using temporal correlations in the Blood Oxygenation Level‐Dependent (BOLD) signal, anatomically similar networks can also be elicited in MEG data using a number of linear, nonlinear, static, and dynamic metrics, the most common involving correlations between band‐limited neural oscillatory amplitude [Baker et al., 2014; Brookes et al., 2011, 2012; Hipp et al., 2012; Luckhoo et al., 2012; Marzetti et al., 2013; O'Neill et al., 2015; de Pasquale et al., 2010]. Using the RM task to investigate the effects of behavioral salience on these networks in healthy volunteers, we found the most robust effects in a network centered on the bilateral insula and adjacent regions, as well as in a left motor network [Brookes et al., 2012]. While both stimulus types (relevant and irrelevant) elicited an initial decrease in beta amplitude, a phenomenon known as event‐related desynchronization (ERD), followed by an increase above baseline, known as event‐related synchronization (ERS), these effects were significantly enhanced for relevant compared to irrelevant stimuli. The most pronounced effect of stimulus relevance was ERS in the insula network, beginning about 400 ms after stimulus onset.

One possible explanation of the observed biphasic ERD–ERS of event‐related beta‐modulation is that the ERD reflects decreased excitatory–excitatory and increased excitatory–inhibitory reciprocal local connections between excitatory pyramidal and fast‐spiking inhibitory interneurons, resulting in a transition from beta to gamma oscillations, and a reduction in beta amplitude [Kopell et al., 2010, 2000]. Donner and Siegel [2011] propose that such transient modulations of frequency from beta to gamma following a stimulus reflects local gamma‐band processes implicated in the encoding of stimulus features or motor responses. In contrast, they suggest long‐range integrative processes over large networks are associated with increased oscillatory amplitude over a more diverse oscillatory range, including frequencies in the beta range. For instance, they cite evidence from an MEG study using a coherent motion detection task [Donner et al., 2007], in which they found that a slowly rising beta signal following stimulus presentation and localized to frontal and parietal sources was superimposed on a more rapid posterior event‐related beta desynchronization, and associated with greater perceptual accuracy.

Thus, the characteristic biphasic ERD–ERS pattern observed in response to salient task‐relevant stimuli may reflect the summation of a short‐latency transition from beta to gamma, reflecting local encoding processes likely to be implicated in “bottom–up” salience detection, with a more gradual later‐peaking increase in a beta signal generated by the integration of information across remote sites, as would be required, for instance, in demanding perceptual tasks such as the coherent motion detection task used by Donner et al. [2007]. Such a summation would result in a net biphasic ERD–ERS signal, even if the integrative beta generator process began contemporaneously with the local beta‐to‐gamma transition.

Interestingly, while abnormalities of oscillations in schizophrenia have been observed across a wide range of frequencies [Doege et al., 2010; Fujimoto et al., 2012; Grutzner et al., 2013; Stephane et al., 2008; Uhlhaas and Singer, 2010], evidence implicates beta‐band abnormalities specifically in perceptual integration: Uhlhaas et al. [2006] observed that deficits in Gestalt perception in schizophrenia were specifically associated with reduced beta‐band phase synchrony between spatially remote sites, while Sun et al. [2013] using a similar perceptual task, showed reduced correlation between beta‐band oscillations and high gamma band activity in first episode cases of schizophrenia relative to controls.

Given, first, our proposed model of schizophrenia as a disorder of salience processing arising from disruption to an insula‐centered salience network; second, our observation of modulation of event‐related beta by stimulus relevance in the bilateral insula in healthy participants; and third, evidence for beta‐band abnormalities in schizophrenia that are likely to reflect disrupted connectivity resulting in impaired integrative processing, we hypothesized that patients with schizophrenia would show abnormal modulation by task‐relevance of event‐related beta amplitude in the bilateral insula, and, specifically, that the enhanced rising beta signal induced by task‐relevant stimuli that we had observed in the insula in healthy participants would be disrupted in schizophrenia.

## METHODS

### Participants

Twelve patients satisfying criteria for schizophrenia according to the Diagnostic and Statistical Manual of Mental Disorders, 4^th^ edition [American Psychiatric Association, 1994], were recruited from community‐based mental health teams in Nottinghamshire, UK, including the Early Intervention in Psychosis teams. Diagnosis was made in accordance with the procedures of Leckman et al. [1982] and a standardized clinical interview [Liddle et al., 2002]. Patients were in a stable phase of illness, defined as a change of no more than 10 points in their Global Assessment of Function score [American Psychiatric Association, 1994], assessed both 6 weeks prior to and immediately prior to study participation. No patient had a change in antipsychotic, antidepressant, or mood‐stabilizing medications in the 6 weeks prior to the study. The mean Defined Daily Dose of antipsychotics was calculated for all patients [WHO Collaborating Centre for Drug Statistics and Methodology, 2003]. For inclusion in the study, patients had to be between 18 and 50 years old, and have an IQ score of above 70 (measured by the Quick Test [Ammons and Ammons, 1962]). Healthy volunteers were recruited from the community via advertisements to form a control sample, matched groupwise to the patient group for age, sex, and parental socioeconomic status (SES), assessed according to the National Statistics Socio‐Economic Classification [Rose and Pevalin, 2003]. A clinical interview by a research psychiatrist was performed to ensure that the controls were free from current Axis I psychiatric disorders; history of psychotic or neurological disorder; or a history of psychotic illness in a first degree relative. After exclusion of two participants' datasets (one patient and one control) owing to excessive movement artefacts in the MEG data, demographic data (Table 1) from the remaining participants were then rechecked to ensure that there were no statistically significant differences between the groups in mean age, parental SES, or gender composition. As this resulted in groups that were no longer matched for age, the youngest control participant (age 20) and the oldest patient (aged 50) were removed from further analyses, leaving data from 10 patients and 12 controls. Demographic data from these participants is given in Table 1. Of the 10 patients retained in the study, seven were receiving antipsychotic medication, and three were not. All participants gave written informed consent according to the World Medical Association Declaration of Helsinki, and the study was given ethical approval by the National Research Ethics Committee, Leicestershire, United Kingdom.

**Table 1:** Demographic data from patients with schizophrenia and healthy controls who remained in the study after movement artefact rejection

	Patients	Controls
Number of participants	10 (8 male)	12 (8 male)
Mean age (standard deviation)	37 (10.3)	30.3 (6.1)
Mean IQ (standard deviation)	96 (12.7)	104(10.7)
Mean parental SES (standard deviation)	3 (1.6)	2.8 (1.6)
Mean defined daily dose of antipsychotic medication (standard deviation)	0.72(0.55)	n/a

Groups remained matched statistically (differences not significant at *p* < 0.05) for age, gender, and SES.

### Relevance Modulation Task

The RM task is a target‐detection task that has minimal motor response requirements and in which the task‐relevance of the stimuli is manipulated. In each block of trials, there were two types of stimuli: images of butterflies and images of ladybirds, and two types of block: butterflies‐relevant and ladybirds‐relevant. Images of typical stimuli are provided in Supporting Information, Figure S1. Stimuli were presented in eight blocks, during each of which images of butterflies and images of ladybirds alternated regularly. Blocks lasted for 90 s, including instructions, and consisted of 40 stimuli (20 butterfly images, 20 ladybird images), followed by a 30 s rest period during which time the participant was required to look at a fixation cross. Stimulus duration was 800 ms, and to avoid entrainment of oscillations, intertrial intervals were randomly drawn from a Gaussian distribution with a mean of 1930 ms and a standard deviation of 40 ms. Subjects were instructed before each block that either the butterflies or the ladybirds would be the relevant stimuli in that block, and to ignore the intervening irrelevant stimuli. The eight blocks were presented in the pattern: B–L–L–B–L–B–B–L (where B = butterflies‐relevant and L = ladybirds‐relevant). For the butterflies‐relevant blocks, participants were shown a color‐filled line drawing of a target butterfly with a specific shape, inner wing color, and outer wing color at the start of the block and instructed to press a button every time they saw a butterfly that matched the target on all three attributes, while ignoring the interleaved images of ladybirds. In the ladybirds‐relevant condition, an image was a target if there were equal numbers of red and yellow ladybirds. The ladybirds were positioned and oriented randomly on each stimulus presentation and the total number of ladybirds ranged between four and six. Participants were instructed to press a button for a target ladybird image, while ignoring the intervening images of butterflies. For both block‐types (butterflies‐relevant and ladybirds‐relevant) the probability of a target being presented was 0.05, a value set intentionally low so as to ensure close attention to the stimuli while minimizing trials with button presses. All participants were given a full explanation of the task outside the scanner as well as an opportunity to practice. During practice, the stimulus timings were at first controlled by the scanner operator via a button press to advance each stimulus presentation at a slower speed to ensure the participant fully understood the task. The speed was gradually increased and practice continued until the operator was satisfied that the participant understood the task and could perform at full speed. Therefore, there was no set practice time; practice duration was tailored to each participant's learning rate.

### Data Acquisition

MEG data were acquired using the third‐order synthetic gradiometer configuration of a 275 channel MEG system (MEG International Services Ltd., Coquitlam, Canada), at a sampling rate of 600 Hz and using a 150 Hz low pass antialiasing filter. Three head position indicator coils were attached to the participant's head at the nasion, left preauricular, and right preauricular points. These coils were energized sequentially with the participant inside the scanner to allow localization of the head relative to the geometry of the MEG sensor array. Head location was measured, using the three head position indicator coils, at the beginning and the end of data acquisition. Any subject whose head moved more than 8 mm (Euclidean distance) between the beginning and end of the scan was removed from the study.

The surface shape of the participant's head was digitized using a 3D digitizer (Polhemus, Isotrack) relative to the coils. The surface shape of each participant's head and the coil locations were then registered to the head surface extracted from an anatomical MR image, acquired using a Philips 3T Achieva MR system at 1 × 1 × 1 mm isotropic resolution using an MP‐RAGE sequence.

### MEG Data Analysis

MEG data were inspected visually for artefacts and blocks containing excessive interference (for example, caused by the magnetomyogram) were removed (three patients had 1, 3, and 3 bad blocks removed, respectively; two controls had 1 and 2 blocks removed, respectively, resulting in a total of 6% data loss). Data from all target trials and from any trial containing a button press were removed, leaving only data from nontarget trials with no button responses.

One problem in comparing network function in healthy participants with a patient group is that of defining networks in a manner that represents healthy function yet is orthogonal to the contrast between the healthy and patient groups in this study. One solution is to define the networks on an independent sample of healthy participants. For this study, we decided to use the network maps generated by our previous study [Brookes et al., 2012]. These had been derived from data acquired from an independent sample of healthy participants, during three tasks including the RM task. The maps had been generated using a “meta‐ICA” process, in which four initial ICAs were performed on the amplitude envelopes of the concatenated beam formed time courses filtered into four broad frequency bands (theta, alpha, beta, and delta). A second ICA was then conducted on the output from these four band‐specific ICAs. The purpose of this procedure was to generate weighted spatial maps representing between‐band as well as within‐band temporal correlations. Because each spatial map represented one component, they included many voxels that made little contribution to the component, and were likely to represent noise. Visual inspection of the maps suggested that a single threshold of 0.3 would generate anatomically plausible maps for all components. [Beckmann et al., 2005; Seeley et al., 2007; Smith et al., 2009] (see Brookes et al. [2012] for further details). We considered therefore that the weighted and thresholded spatial maps of the bilateral insula network and the left motor network from that previous study were an appropriate yet orthogonal operationalization of the networks relevant to this study.

Anatomical MRIs were segmented to remove the skull and scalp using the brain extraction tool (BET) [Smith, 2002] in the fMRIB software library (FSL) [Jenkinson et al., 2012]. They were then downsampled spatially to an 8 mm isotropic resolution. The two thresholded network maps were then registered to each participant's (8 mm) anatomical space using FLIRT [Jenkinson et al., 2002]. To generate a time course of electrophysiological activity for each network, individual voxel signals were first derived. Scalar beam former weights and corresponding voxel time courses, ${\hat{Q}}_{i}\left(t\right)$, were calculated at the center of all voxels (on the 8 mm grid) spanning the regions of interest defined by the network maps. Covariance was computed within a 1–150 Hz frequency window and a time window spanning the whole experiment in order to minimize covariance matrix error [Brookes et al., 2008]. Regularization was applied to the data covariance matrix using the Tikhonov method with a regularization parameter equal to 4 times the minimum eigenvalue of the unregularized covariance matrix. The forward model was based upon a dipole approximation [Sarvas, 1987] and a multiple local sphere head model [Huang et al., 1999]. Dipole orientation was determined using a nonlinear search for optimum signal‐to‐noise ratio (SNR). Beam former time courses were sign flipped where necessary prior in order to account for the arbitrary polarity introduced by the beam former source orientation estimation. Finally, the network time course, ${\hat{Q}}_{R}\left(t\right)$, was generated based on the ICA network map so that

(1) $${\hat{Q}}_{R}\left(t\right)=\sum_{i}W_{i}{\hat{Q}}_{i}\left(t\right)$$

where i represents the count over all voxels within the thresholded network map, ${\hat{Q}}_{i}\left(t\right)$ represents the beam former projected time course for voxel i, and $W_{i}$ denotes the value of the network map for voxel i. Note that this essentially amounts to a weighted sum of electrophysiological time courses across the thresholded network.

Each of the two resulting network time courses were frequency filtered into 17 partially overlapping frequency bands between 1 and 70 Hz (1–4, 2–6, 4–8, 6–10, 8–13, 10–15, 13–20, 15–25, 20–30, 25–35, 30–40, 45–50, 50–60, 55–65, 60–70). For each band, the amplitude envelope was computed as the absolute value of the analytic signal, which itself was calculated via Hilbert transformation. Sequential application of this technique across all bands yielded a time–frequency (TF) spectrogram showing the time evolution of the amplitude envelope of oscillatory power, for all frequency bands of interest. For each network, these amplitude envelopes were averaged across relevant and irrelevant trials, respectively. Data from relevant trials in butterfly‐relevant and ladybird‐relevant blocks were collapsed together, as were data from irrelevant trials. Thus both irrelevant and relevant trial spectrograms included data from both stimulus types (butterflies and ladybirds), so that the only consistent difference between relevant and irrelevant trials was the task‐relevance of the stimulus, not its visual attributes.

The mean oscillatory amplitude in each band was computed during the resting phase of the paradigm (estimated as the average of the amplitude envelope for each frequency band during a 30 s rest period that followed each block of trials). These mean resting values in each frequency band were first checked to ensure that there were no between‐group differences in spectral power (see S2 for resting spectra), then subtracted from the trial‐averaged TF‐spectrograms, yielding time–frequency difference (TFD) spectrograms showing absolute change in oscillatory amplitude from resting baseline for relevant and irrelevant trials in each of the two networks. The spectrograms were then averaged across participants in the control and the patient groups.

### Statistical Hypothesis Tests

The study hypotheses regarding salience abnormalities in schizophrenia were tested by comparing patient and control time courses of mean beta amplitude in each network (insula and motor) for each type of trial (relevant and irrelevant). In order to avoid overlap with the subsequent trial, only data from the first 900 samples (1500 ms) following stimulus presentation in each spectrogram were analyzed.

In the first set of analyses, data from the spectrograms were averaged over three beta‐bands (13–20, 15–25, and 20–30 Hz) and downsampled using a simple averaging kernel (83 ms window) into 18 50‐sample time bins for each participant. These binned data were then entered into a repeated‐measures ANOVA, with three within‐subjects factors (network: insula and motor; relevance: relevant and irrelevant; time: 18 time‐bins), and with diagnostic group as a between‐subjects factor (group: patients with schizophrenia and healthy controls). We operationalized the strength of the biphasic ERD–ERS pattern as the extent to which the time series could be fitted by a cubic polynomial with a negative cubic term and a positive quadratic term. Such a function will have an early trough (to fit the ERD) and a later peak (to fit the ERS). Where an ANOVA yielded a statistically significant effect of time, polynomial contrasts were conducted to test the statistical significance of the cubic and quadratic terms, and the signs of the coefficients checked. Where ANOVAs yielded statistically significant interactions (*p* < 0.05) of interest, follow‐up ANOVAS with the interacting factors separated were carried out in order to interpret the interaction.

### Permutations Tests

As the number of participants in each group was small and the number of variables was large, some significant violations of the assumption of normally distributed residuals were anticipated. Therefore, for each ANOVA, instead of using the standard *F* distribution to compute *p* values for the *F* values output by the ANOVAs, the distribution of *F* values under the relevant null hypothesis was computed using a resampling method. Ten thousand iterations were performed, and the *p* value for each *F* computed as the frequency of *F* values exceeding that observed. For between‐group effects, the null hypothesis was that groups were drawn from the same population. Null datasets were constructed by randomly drawing, without replacement, groups representing the sizes of the healthy and patient groups, respectively, from values from the whole sample. *F* values were then computed for between‐group effects for each of these randomly drawn sets of groups. For the within‐subject factor, relevance, the null hypothesis was that there was no difference between relevant and irrelevant trials. Null datasets were therefore drawn for each participant by taking their pairs of data (relevant and irrelevant) and equiprobably either reversing or retaining, the order of the conditions. The same method was used for the within‐subjects factor, network. For the within‐subjects factor time, the null hypothesis was that there would be no systematic evolution of the signal amplitude over time, and nor any systematic polynomial fit. In order to preserve the mean value of the amplitude differences between consecutive time bins, the step‐changes between each bin value were permuted rather than the data values themselves. The mean value of the data series was assigned to the first time‐bin of the null time series, then for each subsequent value in the time series, a step‐change from the original series, randomly selected without replacement, was added to the previous value.

### Modeling Two Beta Signals

In the second set of analyses, we tested our hypothesis that event‐related beta‐modulation reflects two different, superimposed oscillatory patterns, namely a reduction of beta amplitude in local populations involved in encoding processes, and a rising beta signal implicated in long‐range integrative processes. We modeled the beta suppression associated with local encoding by an early negative‐going curve, and the beta signal associated with long‐range interactions with second curve, postulated to be positive‐going, as illustrated in Figure 3. Both curves were modeled by the two‐parameter Weibull probability density distribution:

(2) $$f\left(T\right)=\frac{b}{a}{\left(\frac{T}{a}\right)}^{b‐1}e^{‐{\left(T/a\right)}^{b}}$$

This distribution is useful for modeling time courses in which a variable rises to a peak then decays to baseline; *a* is the scale parameter >0 that determines the width of the peak, and *b* is the shape parameter <0 that determines its symmetry. Both local and integrative components were modeled by Weibull functions, the function modeling the integrative component being constrained to have a later peak than the function modeling the local component. The local beta component was modeled by a Weibull function with a scale parameter constrained between 283 and 450 ms, and a shape parameter of 3 (approximately symmetrical). The integrative beta component was modeled by a second Weibull function with a scale parameter constrained between 500 and 1700 ms (300–1020 samples), with a shape parameter constrained to lie between 2 (slight negative skew) and 4 (slight positive skew). The parameters were chosen so as to bracket best‐fits for all participants. Two curves at a time (one local, one integrative) were entered as predictors into a series of general linear models for each participant's beta time course in each condition in each network, the curve parameters being iteratively incremented for each model. For each participant, the model with the best fit (smallest sum of squared residuals) was selected, with the additional constraint that the sign of the coefficient for the local beta curve was negative, thus modeling the hypothesized negative deflection. The sign of the coefficient for the integrative beta curve was free to vary. For each participant, the best‐fitting pair of Weibull functions was selected, and the AUC (area under the curve) for local and integrative models was then taken to quantify its respective beta component, and the location of its peak recorded as the latency of the component.

AUCs and peak‐latencies were then analyzed, as with the time‐bin data, in a mixed‐model ANOVA with network, beta‐type, and relevance and network as within‐subject variables and group as a between‐subject variable, with follow‐up analyses on separate factors carried out to interpret any interactions. Again, the distribution of *F* values under the relevant null hypothesis was determined by resampling over 10,000 iterations, and the *p* value evaluated as the proportion of *F* values exceeding those observed.

### Long‐Range Integrative Function

As a final check on the concept underlying the partition of beta power into “local” and “integrative” components, we tested the hypothesis that if our putative integrative beta values were involved in long‐range integrative function between networks, we would see a positive correlation between insula integrative beta and the gamma signal assumed to reflect local neural activity in a remote region engaged during the task, at least in healthy control subjects in the relevant condition. We therefore computed the gamma spectrogram (8 band overlapping bands: 25–35 Hz, 30–40 Hz, 45–50 Hz, 50–60 Hz, 55–65 Hz, 60–70 Hz) for each trial type (relevant an irrelevant) in a primary visual network also defined in our previous study [Brookes et al., 2012] and compared the correlations, for each subject, over their 900 averaged poststimulus sampled time‐points, between the predicted value of their integrative beta signal from the insula network at each time point and the power in each of the 8 gamma band frequencies at each time point. Fisher‐transformed correlation values were then entered into a three‐way mixed ANOVA (2 levels of relevance: relevant and irrelevant; 8 levels of visual gamma frequency; 2 levels of diagnostic group).

## RESULTS

### Behavioral Data

Task accuracy was measured for each participant as *d′* score (*z*‐transformed proportion of targets correctly identified minus *z*‐transformed proportion of nontargets misidentified). For healthy control participants, mean *d′* score was 3.15 (SD = 1.12), equivalent to a mean percentage accuracy of 99.9%, while for patients, mean *d′* score was 1.95 (SD = 1.3), equivalent to a slightly lower mean percentage accuracy of 97.4%. For healthy control participants, mean reaction time (RT) was 804 (SD = 181), and for patients, mean RT was 798 (SD = 108). Between‐group comparisons of *d′* scores and RTs using independent samples *t*‐tests revealed that patients were significantly less accurate in the task, *t*(20) = 2.330, *p* = 0.03, but that there was no significant difference between groups in mean reaction times (*p* = 0.9).

### Spectrograms

**Figure 1:**
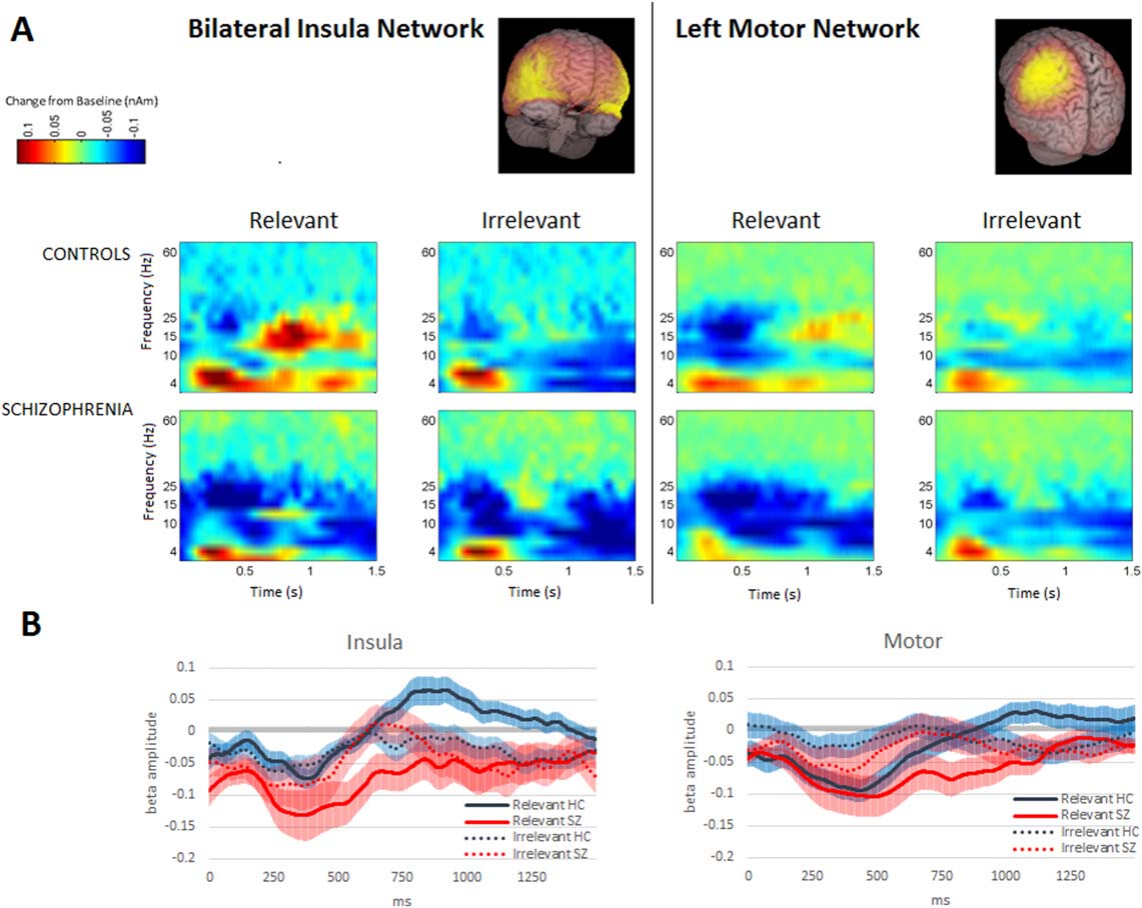
(A) Time frequency difference spectrograms representing oscillatory activity (relative to resting baseline) for the 2 s following presentation of a stimulus, for each network, group, and relevance condition. Time is on the horizontal axis, 0 corresponds to the stimulus presentation, and stimulus duration was 800 ms. Frequency is plotted on the vertical axis. Healthy participants, during relevant trials, show a marked period of beta ERS extending to a level above resting baseline in the insula network after a brief period of beta ERD; in the motor network during relevant trials, a similar pattern is observed. These effects are attenuated in irrelevant trials. Patients, in both networks, during both relevant and irrelevant conditions, show more conspicuous and prolonged beta ERD, and the suprabaseline insula ERS effect visible in controls in relevant trials appears to be absent. (B) Mean change from resting baseline of oscillatory amplitude in the beta‐band (13–30 Hz) in each network during the first 1500 ms of each trial. Controls are shown as a solid black line, patients as a red line (white in the print edition). Relevant trials are indicated by a solid line, irrelevant by a dotted line; standard errors are shown as transparent colored borders. [Color figure can be viewed in the online issue, which is available at http://wileyonlinelibrary.com.]

Spectrograms created from beam formed MEG data, spatially filtered by bilateral insula and left motor network masks, respectively, are shown in Figure 1, together with time courses of the beta‐band amplitude, and are in agreement Brookes et al. [2012].

### Statistical Tests

#### Modeling by time‐series

First, to establish whether, overall, the pattern of significantly enhanced beta ERD followed by ERS in relevant as compared with irrelevant trials that we observed in our earlier study [Brookes et al., 2012] in these networks, was replicated to a statistically significant degree in this study, we ran an initial set of ANOVAs on beta‐band data from whole group, irrespective of diagnosis, with network, time, and relevance as repeated within‐subjects variables (no between‐subjects factors). Test statistics from these initial ANOVAs are shown in Table 2, and confirmed a significantly biphasic ERD–ERS (averaged over both networks and conditions) that was significantly more marked in the relevant than in the irrelevant condition. As with our earlier study, these effects were present in both networks, as indicated by significant *F* values for the time and time × relevance factors, and with significantly negative cubic and positive quadratic terms for the polynomial contrasts, as predicted in our hypothesis.

**Table 2:** Statistics for ANOVAs testing within‐subjects effects in the pooled sample

Network	Factor(s)	Effect, *F*	Polynomial contrast, *p*	Cubic term, *F*	Quadratic term, *p*	*p*	*p*
Both	Time	18.060	<0.001	49.797	<0.001	<0.001	<0.001
	Time × Relevance	10.399	<0.001	31.457	<0.001	<0.001	<0.001
Insula	Time	12.845	<0.001	38.511	<0.001	<0.002	<0.001
	Time × Relevance	4.748	<0.002	14.559	<0.001	<0.001	<0.001
Motor	Time	14.416	<0.001	29.070	<0.001	<0.001	<0.001
	Time × Relevance	10.971	<0.001	25.954	<0.001	<0.001	<0.001

*F* values are given for each effect (d*f* = 17,357), and for the cubic polynomial contrast (d*f* = 1,21). Cubic terms were all negative and quadratic term were all positive. All *p* values were computed by resampling.

**Figure 2:**
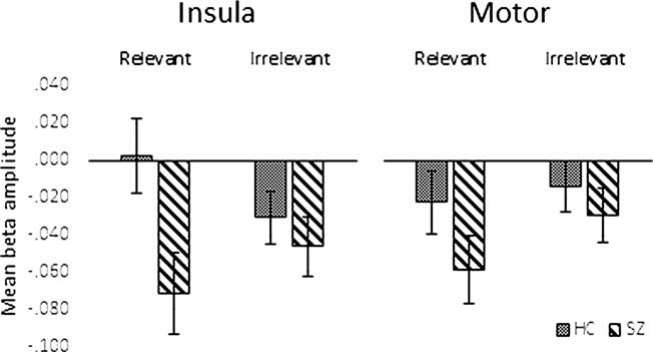
Mean beta amplitude values averaged across all time bins for each condition in each network, for each group. A significant Group × Relevance interaction in the insula indicated that the patients with schizophrenia showed a less marked modulation by relevance than healthy controls, showing marked mean beta desynchronization in both conditions, while a significant Group × Relevance × Network indicated that this effect of diagnosis was significantly less marked in the motor network. Error bars represent 95% confidence intervals.

We then tested our main study hypothesis that modulation of the beta signal by stimulus‐relevance in these networks would be disrupted in schizophrenia. Test statistics are shown in Table 3 (upper panel). As can be seen from the spectrograms and line plots Figure 1, the effects of relevance on mean beta amplitudes differed between the two groups, and this effect of diagnosis differed significantly between networks. In the insula, the effect of relevance in the healthy control subjects was significantly greater than in the patients. While in healthy controls, task‐relevant stimuli raised mean beta amplitudes above those in irrelevant trials to the extent of reaching or exceeding baseline, in patients, mean change from baseline did not differ significantly between trial types, and was significantly below baseline in both trial types. However, in contrast, in the motor network, there were no differences between patients and controls in the extent to which beta amplitude was modulated by relevance. The findings remained robust when age was included as a covariate, and age was not a significant predictor of beta amplitudes. Mean beta amplitudes for each group, condition and network are shown graphically in Figure 2. Groups also differed significantly in their modulation of beta over time by relevance (relevance × time × group interaction), indicating, as hypothesized, altered modulation by relevance of the time course of event‐related beta in schizophrenia. The nature of this modulation is delineated in the analysis reported below.

**Table 3:** Statistics for mixed‐model ANOVAs showing omnibus test as well as tests done to follow up significant interactions

*Effects on mean beta amplitudes*					
Network	Factor(s)			*F*(d*f*)	*P*
Both	Network × Relevance × Group			6.845 (1,20)	<0.02
	Network × Relevance × Group (with age as covariate)			5.347 (1,19)	<0.05
	Relevance × Time × Group			3.106 (17,340)	<0.05
	Relevance × Time × Group (with age as covariate)			2.107 (17,323)	<0.05
Insula	Relevance × Group			9.676 (1,20)	<0.006
	Relevance × Group (with age as covariate)			5.588(1,19)	<0.05

*Effects on beta‐types*					
Beta‐type	Network	Group	Factor(s)	*F*(d*f*)	*P*
Both	Both	Both	Beta‐type × Relevance × Group	5.877 (1,20)	<0.02
Integrative	Both	Both	Relevance × Group	8.727 (1,20)	<0.007
Integrative	Insula	Both	Relevance × Group	12.160 (1,20)	<0.002
Integrative	Insula	Healthy	Relevance	6.176 (1,11)	<0.04
Integrative	Insula	Patients	Relevance	5.826 (1,9)	<0.05

Upper panel shows tests on mean beta amplitudes; lower panel shows tests on beta‐type (local and integrative beta). All *p* values were computed by resampling.

#### Modeling by beta‐type

**Figure 3:**
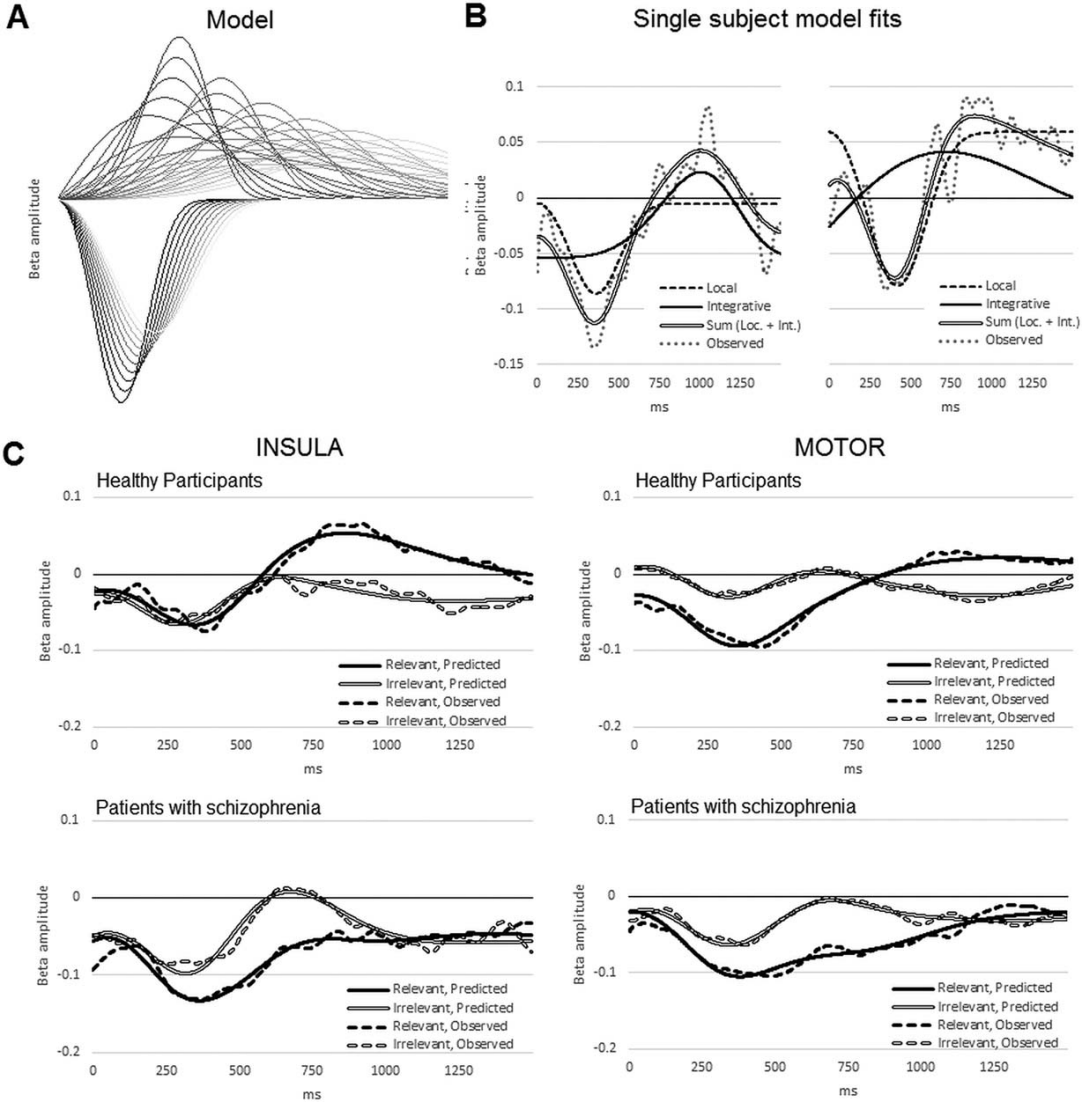
(A) Samples from the series of Weibull distributions used to fit each participant's data. Downward deflecting curves represent desynchronization of local beta, the upward deflecting curves represent integrative beta. (B) Examples of a fitted model for two participants. The dashed curves represent the fitted local beta model, and the solid curves the fitted integrative beta model. The summed curves are indicated by a double line and the participant's observed data as a dotted line. The left‐most example shows a fitted integrative beta curve with a fairly long latency and narrow width; the right‐most example shows a fitted integrative beta curve with shorter peak latency and larger width. Both sum to form the characteristic beta ERD–ERS biphasic pattern. (C) Mean fitted curves for each group, in each condition, in each network. Mean model curves are shown as continuous lines, the mean observed data as a dashed line.

We then investigated the effects of relevance and diagnosis on our modeled local and integrative beta measures (AUCs for each postulated beta type) respectively. The modeling process is illustrated in Figure 3.

**Figure 4:**
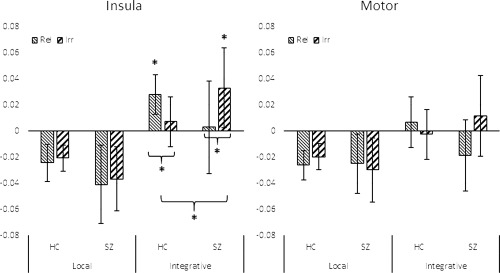
Effects of integrative beta (AUC) by network, condition, and group. Statistically significant contrasts (*p* < 0.05) are shown by an asterisk; error bars represent 95% confidence intervals. In the insula, healthy control participants showed integrative beta values that were significantly greater in the relevant condition than in the irrelevant condition, and significantly greater than zero. Patients actually showed a reversal of this pattern, showing significantly greater integrative beta in the irrelevant than relevant condition. The Group × Relevance contrast was also statistically significant. This was reflected in the motor network as a trend, but did not reach statistical significance. There were no significant effects of group or relevance on local beta.

There was a significantly greater effect of relevance and group on integrative beta than on local beta. Test statistics from these ANOVAs are shown in Table 3 (lower panel), and means are graphed in Figure 4. For local beta, there were no significant effects of diagnosis on the effect of relevance, whereas for integrative beta, the effect of relevance differed between groups. In the insula, both groups showed significant effects of relevance, but in opposite directions. For the controls, as expected, integrative beta was significantly higher in the relevant than in the irrelevant condition, whereas for patients, the opposite was the case: integrative beta was significantly higher in the irrelevant condition than the relevant. As with mean beta, in the motor network effects of diagnosis on modulation by relevance of integrative beta did not reach statistical significance (*p* value from resampling = 0.221). There were no significant effects of relevance, network, or group on peak latencies for either beta‐type.

### Insula Integrative Beta and Visual Gamma

**Figure 5:**
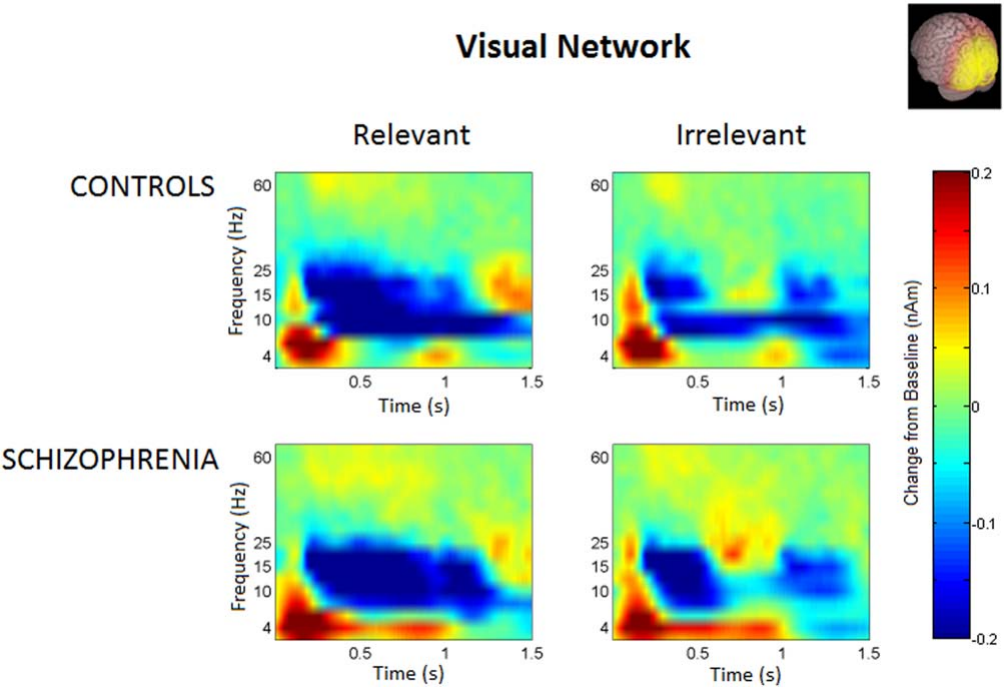
Time frequency spectrograms for the visual network, by group, and relevance condition. [Color figure can be viewed in the online issue, which is available at http://wileyonlinelibrary.com.]

**Figure 6:**
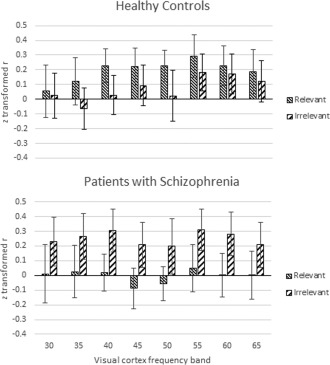
Correlations between insula integrative beta and visual gamma. The height of the bars shows the mean *z*‐transformed correlation between the inferred insula integrative beta signal and 8 overlapping gamma band oscillations from primary visual cortex, centered on the frequencies shown. Error bars are standard errors. Overall, the correlations were significantly positive, with correlations for relevant trials in the healthy group being statistically significant at *p* = 0.008.

Spectrograms spatially filtered by the visual network mask are shown in Figure 5. In the three‐way ANOVA testing the correlations between the predicted insula integrative beta time course for each trial type, and visual gamma in eight frequency bands, the correlation, across all diagnoses and trial types was significantly positive, *F*(1,20) = 7.481, *p* = 0.013. The relevance × group interaction trended to significance, *F*(1,20) = 3.858, *p* = 0.064, with the correlation tending to be stronger in the relevant condition for healthy controls, and in the irrelevant condition for patients. For the healthy controls, the correlation between visual gamma and insula integrative beta was significantly positive, *F*(1,11) = 10.284, *p* = 0.008 for relevant trials, and remained significantly positive when averaged across both conditions, *F*(1,11) = 6.417, *p* = 0.028, but did not reach significance for irrelevant trials. For patients, the effect was larger for irrelevant than for relevant trials, but neither was significant either separately or when averaged across conditions. There were no significant effects of specific gamma frequency. These findings are shown graphically in Figure 6.

As a check on the specificity of this result, we used the same curve‐fitting procedure to compute integrative beta values for the visual beta band, and repeated the analysis on data from healthy controls for relevant trials but with visual integrative beta as the predictor of power in the 8 gamma bands in each network in turn (insula, motor, and visual). There was no significant correlation between visual integrative beta and gamma in any of the three networks (*F* < 1 in each case), including the relationship between visual integrative beta and visual gamma, that is, gamma in the same network. We also tested the correlation between insula integrative beta and gamma in the same (i.e., insula) network and also found no correlation (*F* < 1). This supports the interpretation that, as we hypothesized, insula integrative beta is implicated specifically in modulating long‐range gamma band activity, for instance, in a cortical network implicated in processing sensory stimuli.

### Controlling for Possible Medication Effects

Finally, to explore whether medication was likely to be a contributory explanation for these results, the effect of diagnosis on beta modulation by relevance was tested including only the three patients who were not currently receiving medication. In the time‐series analysis, the Group × Relevance effect remained significant in the Insula, *F*(1,13) = 6.301, *p* < 0.05, as did the Group × Relevance effect in the insula on integrative beta *F*(1,13) = 5.720, *p* < 0.05.

## DISCUSSION

Our results support our hypothesis that modulation by task‐relevance of event‐related beta‐band oscillations in the insula would be disrupted in schizophrenia. They also support our specific prediction that the rising beta signal postulated to represent long‐range integrative processes [Donner and Siegel, 2011], which we expected to be enhanced by task‐relevant stimuli in healthy controls, would be modulated abnormally by task‐relevance in patients, in line with both the aberrant salience hypothesis and the dysconnectivity hypothesis. Healthy controls showed greater mean beta‐amplitude for task‐relevant trials as compared with irrelevant trials, an effect not observed in patients, and this difference between patients and controls was significantly more pronounced in the insula than in the motor network. Moreover, in healthy controls, as expected, the rising beta‐signal postulated to reflect integrative processes was significantly enhanced in response to task‐relevant images compared to irrelevant images, while in patients, this signal was actually significantly greater for irrelevant images than for task‐relevant images.

The early short‐latency beta decrease postulated to be associated with local encoding was not significantly modulated by relevance, and did not differ between the groups. We also found a significantly greater effect of diagnosis in the insula than in the motor network on overall modulation of beta‐amplitude by task‐relevance. These findings are consistent with the proposal that the insula plays a key role in integrating networks required to deal with salient events.

The observed reversal of the normal modulation in the insula of integrative beta by task‐relevance in patients with schizophrenia is noteworthy, and may reflect misplaced attribution of salience to nonsalient events, and thus relate to the aberrant salience model of delusions and hallucinations proposed by Kapur [2003]. Moreover, the specificity of this effect to the insula network is consistent with the abnormal salience network hypothesis of Palaniyappan and Liddle [2012].

The finding of significant overall positive correlations between insula integrative beta and visual gamma supports the interpretation of this beta signal as implicated in mediating integrative processes between networks. We anticipated that strength of the rising integrative beta signal in the insula would be reflected in increased local gamma encoding processes in the visual cortex, particularly when the stimulus was relevant and ongoing visual processing would be required before the stimulus could be dismissed. This was supported by our findings, and was strongest as we anticipated (and statistically significant), in the healthy controls while engaged in processing relevant stimuli, although the difference between the effect in relevant stimuli and irrelevant did not reach statistical significance. In the light of our findings of reduced integrative beta in the insula in patients in relevant conditions, this suggests that a reduced insula‐integrative beta signal in schizophrenia may result in impoverished gamma oscillatory processes in visual cortex where these are required for adequate evaluation of a relevant stimulus.

An interpretation of our findings in terms of dysfunctional integrative processes at the beta frequency in schizophrenia is consistent with the study by Uhlhaas et al. [2006] demonstrating diminished high beta (20–30 Hz) phase coherence between spatially remote sites in schizophrenia, as well as with the body of literature characterizing schizophrenia as a dysconnectivity disorder [Fitzsimmons et al., 2013; Fornito et al., 2012; Friston and Frith, 1995]. Our findings regarding disturbed integrative oscillatory activity in the insula are thus consistent both with the large body of evidence from systems neuroscience for subtle but extensive abnormalities of brain function in schizophrenia and with the evidence from cellular neuroscience for disordered neurotransmission.

The observation that the three cases not on medication showed significant abnormalities of beta modulation by relevance similar to those seen in the medicated cases, together with evidence from other studies indicating that oscillatory abnormalities in schizophrenia are present in unmedicated cases [Allen et al., 2011; Boutros et al., 2008; Gallinat et al., 2004; Sun et al., 2013] and first‐degree relatives [Hong et al., 2008], suggest that the abnormalities observed in our study are unlikely to be accounted for by the effects of medication. Nevertheless, further investigation to examine the effects of medication on the oscillatory abnormalities observed in this study is warranted.

We conclude that beta oscillations play at least two important roles in processing of task stimuli in healthy subjects, the second of which is disrupted in schizophrenia. First, both relevant and irrelevant task stimuli induce a rapid but transient phasic reduction in beta amplitude relative to baseline, probably due to the retuning of local excitatory–inhibitory encoding circuits from the beta to the gamma range. Second, in response to task‐relevant stimuli, there is enhancement of a more gradual phasic increase in beta amplitude relative to baseline, postulated to represent a beta signal implicated in integrating information across widely distributed brain regions. Together, these two opposing beta effects (early suppression of local beta in favor of gamma; more gradual increase in long‐range beta integrative signal) sum to produce an initial net beta amplitude reduction (beta ERD) followed by a sharp increase (beta ERS). In schizophrenia, we conclude, the local beta encoding signal, reflected in the early beta desynchronization, is not abnormal. However, in the insula network, the integrative beta signal showed an attenuated response to task‐relevant stimuli while conversely actually showed a significantly increased response to irrelevant stimuli. Taken together with robust evidence for grey matter deficits in the bilateral insula, this suggests that in patients with schizophrenia a faulty salience network fails to signal the salience of task‐relevant stimuli, resulting in disruption to the integrative processes required for efficient evaluation of stimuli and appropriate response selection. Such disruption could lead both to failure to recognize what is relevant, and to attribution of salience to what is irrelevant, thereby contributing to the distortion of reality and the disorganization and impoverishment of mental activity that occur in schizophrenia.
